# Impactul afecțiunilor metabolice asupra tulburărilor affective

**Published:** 2014

**Authors:** Ladea Maria, Barbu Cristina Maria, Roșu Dan Petru

**Affiliations:** * Spitalul Clinic de Psihiatrie “Prof. Dr. Al. Obregia”, București, România

**Rezumat**

**Introducere:** Datele din literatura de specialitate sugerează existența unei corelații între depresie și diabetul zaharat (DZ) dar relația de cauzalitate nu este înca clarificată. Depresia este asociată cu hiperglicemie și cu un risc crescut de complicații ale diabetului, astfel o ameliorare a depresiei se asociază cu un control mai bun al glicemiei.

**Obiective:** Obiectivul acestui studiu este de a prezenta aspectele clinice ale depresiei și opțiunile terapeutice la pacienții depresivi cu diabet zaharat asociat.

**Material și metodă:** Studiu naturalistic, observațional în care au fost incluși 21 de pacienți cu vârste cuprinse între 30 si 59 ani, diagnosticați cu Tulburare Depresivă Majoră conform Manualului de Diagnostic și Statistică a tulburărilor mentale, Ediția IV Revizuită (DSM IV-TR) și DZ tip I sau II, internați în ultimele 12 luni. Simptomatologia depresivă a fost evaluată folosind scala Montgomery-Asberg pentru evaluarea depresiei (MADRS) care a fost aplicată la internare și la externare. Diabetul zaharat a fost evaluat prin monitorizarea glicemiei a jeun și a hemoglobinei glicozilate. Complicațiile diabetice au fost obiectivate prin intermediul explorărilor clinice și de laborator. Toți pacienții au primit tratament antidepresiv (venlafaxină sau sertralină) și tratament hipoglicemiant (metformin sau insulină) conform prescripției specialistului.

**Rezultate:** Din totalul pacienților, 63% au fost de sex feminin, iar vârsta medie a fost de 47.4 ani. Durata medie a spitalizării a fost de 26 zile. Scorul mediu MADRS la internare a fost de 27.6. Valorile medii ale glicemiei a jeun la internare au fost de 178 mg/dl, iar apoi au scăzut la externare concomitent cu îmbunătățirea simptomatologiei depresive. Prevalența DZ tip I a fost de 24%, cu o rată mai mare a complicațiilor diabetice comparativ cu lotul pacienților cu DZ tip II. La pacienții cu DZ tip I valorile medii ale MADRS la internare au fost semnificativ mai mari comparativ cu pacienții cu DZ tip II. Pacienții cu DZ tip I tratați cu venlafaxină au avut o mai bună ameliorare a simptomelor depresive comparativ cu pacienții care au primit sertralină. Dozele medii de antidepresive folosite la pacienții cu DZ tip I au fost mai mari comparativ cu pacienții cu DZ tip II. Perioada totală de spitalizare a fost mai mare la pacienții cu DZ tip I. La pacienții cu DZ tip II răspunsul la tratamentul antidepresiv, evaluat prin scala MADRS la externare, a fost asemănător în cazul venlafaxinei și sertralinei.

**Concluzii:** Depresia asociată cu DZ tip I este mai severă. La acești pacienți este nevoie de doze mai mari de antidepresive și o durată de spitalizare mai mare. Un răspuns favorabil a fost observat la pacienții cu DZ tip I și complicații diabetice preexistente tratați cu venlafaxină comparativ cu același profil de pacienți tratați cu sertralină.

**Cuvinte-cheie:** depresie, diabet, antidepresiv

